# Barriers to access and organization of primary health care services for rural riverside populations in the Amazon

**DOI:** 10.1186/s12939-020-01171-x

**Published:** 2020-07-31

**Authors:** Luiza Garnelo, Rosana Cristina Pereira Parente, Maria Laura Rezende Puchiarelli, Priscilla Cabral Correia, Matheus Vasconcelos Torres, Fernando José Herkrath

**Affiliations:** 1 Instituto Leônidas e Maria Deane, Fundação Oswaldo Cruz, Rua Teresina, 476, Adrianópolis, Manaus, Amazonas 69057-070 Brazil; 2 Programa de Pós-Graduação em Condições de Vida e Situações de Saúde na Amazônia, Instituto Leônidas e Maria Deane, Fundação Oswaldo Cruz, Rua Teresina, 476, Adrianópolis, Manaus, Amazonas 69057-070 Brazil

**Keywords:** Rural population, Primary health care, Delivery of health care, Health equity

## Abstract

**Background:**

The ways of life in the Amazon are diverse and not widely known. In addition, social inequities, large geographic distances and inadequate health care network noticeably limit access to health services in rural areas. Over the last decades, Brazilian health authorities have implemented fluvial mobile units (FMU) as an alternative to increase access and healthcare coverage. The aim of the study was to identify the strategies of access and utilization of primary health care (PHC) services by assessing the strengths and limitations of the healthcare model offered by the FMU to reduce barriers to services and ensure the right to healthcare.

**Methods:**

Qualitative and ethnographic research involving participant observation and semi-structured interviews. Data collection consisted of interviews with users and health professionals and the observation of service organization and healthcare delivered by the FMU, in addition to the therapeutic itineraries that determine demand, access and interaction of users with healthcare services.

**Results:**

Primary care is offered by the monthly locomotion of the FMU that serves approximately 20 rural riverside communities. The effectiveness of the actions of the FMU proved to be adequate for conditions such as antenatal care for low-risk pregnancy, which require periodic consultations. However, conditions that require continued attention are not adequately met through the organization of care established in the FMU. The underutilization of the workforce of community health workers and disarrangement between their tasks and those of the rest of the multi-professional team are some of the reasons pointed out, making the healthcare continuity unfeasible within the intervals between the trips of the FMU. From the users’ perspective, although the presence of the FMU provides healthcare coverage, the financial burden generated by the pursuit for services persists, since the dispersed housing pattern requires the locomotion of users to reach the mobile unit services as well as for specialized care in the urban centers.

**Conclusions:**

The implementation of the FMU represents an advance in terms of accessibility to PHC. However, the organization of their activity uncritically replicates the routines adopted in the daily routine of health services located in urban spaces, proving to be inadequate for providing healthcare strategies capable of mitigating social and health inequalities faced by the users.

## Background

Health and social inequalities are recurrent in Brazil, but they are more severe in rural populations as medical assistance only covers about 40% of the attempts to access health assistance in 1 year. Of those who do seek care, almost half give up due to the long waiting time. Furthermore, the per capita income of residents of Brazilian rural areas is two times lower than the average income in cities and the majority dependent on public health services [1].

The Northern region, with an area equivalent to that of the Brazilian Amazon, has low income levels and the lowest municipal human development indexes in the country. The effects of poor health coverage are more critical due to social inequalities and the lack of public policies geared to meet local needs, negatively influencing the quality and effectiveness of services available to this population who heavily depend on the public health system [2, 3]. Health care provision is limited in this region and a great number of people have never had access to dental care (16.6% of the population) [4, 5]. Among the geographic regions of Brazil, the North has the lowest number of doctors (1/1000 inhabitants) and the greatest disparity between availability of these professionals in large and medium cities (2.5/1000) compared to rural areas (0.4/1000). This rate is almost three times lower than the one found in large cities in the South (7.1/1000) and about four times lower than the distribution of doctors in the inland area of the Southeast region (1.7 / 1000) [6].

As a result, routine primary care such as antenatal care has recurrent levels of insufficiency and inadequacy. Assessment studies of antenatal care have been conducted in Brazil since 2004, showing that the states in the Northern region had the lowest adequacy of antenatal care compared to other Brazilian regions [7]. Despite advances in maternal and child health in recent years in the country, antenatal care in the northern region is still inadequate [8–10]. Recent studies found that 60% of pregnant women who receive antenatal care with insufficient levels of adequacy and limited infrastructure availability. In the Northern region, these percentages of the quality of antenatal care offered in the primary health care (PHC) network were even lower. The states of Amazonas and Pará offered the lowest rate of adequate antenatal care [11, 12]. The worst income conditions, lowest municipal human development indexes and lowest PHC coverage were also found to be inadequate in these states (< 70%) [12].

Child health is also poor in rural areas and comprehensive studies for the whole Amazon are also scarce. A study conducted in a rural area of the northern region that evaluated the health conditions of children under the age of 2 years, including nutritional status, physical-motor development and health problems in the last month, found 59.4% of the children examined presented poor health status; of these, 17.3% had low weight and height-for-age. Regarding health problems, 18.5, 14.3 and 32.6% of children < 6 months, 6–11 months and 12–23 months, respectively, presented with diarrhea 15 days prior to data collection [13].

The literature shows a lack of information, not only concerning the health of rural Amazonian populations, but also the characteristics of regional social diversity, whose ways of life have a strong influence on the perception of the health-disease process and access to and use of health services. The characterization of ‘rural’ and its populations is a complex task in the Amazon either due to the wide variation in the ways of life of the groups or to the limitation of indicators adopted by the official departments, such as employment and income, to account for the diversity of economic relations in the Amazon. Considering rural families as peasants is also questionable, since most of them develop agricultural activities, that are seasonal and intermittent, primarily targeted for domestic consumption, and they generate little surplus for trading [14, 15]. The economically active rural population usually has multiple activities: they extract wood, chestnuts, vine and other products from the jungle, practice fishing, hunting, and build and drive boats, construct houses, and carry out other typically Amazonian activities for family subsistence. These activities are intrinsically linked to an economic history associated with extractivism rather than agriculture, shaped by diverse labor processes which vary throughout the seasons, which do not easily fit the notion of single or main family occupation in the urban context [15].

Another way to characterize the Amazon social diversity has been to identify the origin and ethnic identity (caboclos, mestizos) and/or attribute certain geographical (riverines) characteristics. Criticism of the designation of ethnic origin addresses the polysemic and unenlightening character of the term as well as its association with the historical fact of forced miscegenation that informs nothing about the current sociological condition of these individuals in addition to carrying a negative semantic load that suggests a subordinate and depreciated social position [14].

The also-criticized term ‘riverside’ used to identify these populations prioritizes a geographical-spatial distribution along river banks, reducing the way of life of rural Amazonian populations to a specific aspect – their place of residence – which excludes them from other sociopolitical dimensions as well as from social groups that do not live along river banks. It also obscures one of the most important features of the Amazonian way of life that is mobility [16]. Despite the great distances, absence of roads, lack of regular transport and significant geographical barriers that are typical in the region, the movement of people and goods transiting between the countryside and cities is intense. These travel routes through the water follow the hydrological cycles of drought and flooding as the configuration of the river changes seasonally. Flood periods allow interfluves navigation, shortening distances, increasing mobility and contributing to the establishment of multiple relationships that blur the distinction between rural and urban areas [17, 18].

Authors such as Lima and Pozzobon [14] have also pointed out the need to recognize the socioeconomic and historical changes in the region, such as the expansion of the presence of the Brazilian national state – with emphasis on health and education – and the emergence of environmental activism, particularly of actions related to sustainability and local development, that seeks alliances with traditional populations of the region. Such initiatives deepen the intertwining between the subsistence economy supported by the extraction of forest resources and the so-called green economy that is seen as an alternative to promote sustainability. In addition to contributing to the valorization of historically discriminated populations, the environmentalist approach provides new basis for understanding Amazonian social diversity by characterizing it by the degree of sustainability of the use of environmental resources in an attempt to overcome the analysis oriented by the financial market and social exclusion, as usual.

Based on this premise, the authors proposed a classification of Amazonian social groups according to the sustainability gradient of occupation of the environment, considering the pressure on the ecological sustainability of the forest and knowledge of the ecosystem that provide productive processes with low environmental impact. The group that has received the designation of ‘small traditional producers’, whose environmental sustainability is classified as average, is the one of interest in this article [14]. They are inhabitants of rural, demographically dispersed areas, whose labor activities are supported by limited technological resources that prevent intensive exploitation of natural resources available in their territories, even though the purchase of manufactured goods acquired in the nearest cities is significant. The housing pattern can be heterogeneous, with families who own a house in the rural area and one near the plantation and/or extractive sites and still have a home or relatives in the city, configuring a support network for high mobility that expresses continuity between river, forest and city, as well as multiple relations with various physical and social spaces that are typical of inland living [16].

From a cultural standpoint, these populations are recognized as *quasi-ethnic groups* who share a common worldview, have distinct habits and behaviors from urban residents and extensive knowledge of the forest that is transmitted orally from one generation to another, although they do not have a cohesive cosmology that supports knowledge of nature, as it occurs with indigenous groups [14, 16].

The difficulty in accurately characterizing the study population highlights its multi-ethnic origin and ambiguous and paradoxical character, which was characterized by Harris [19] as being multi-diverse, simultaneously cosmopolitan and modern, traditional and regional. These singularities create specific health needs that should be considered by official health policies.

Over the last decades, Brazilian health authorities have implemented the National Policy for Primary Health Care (PNAB, Portuguese acronym for *Política Nacional de Atenção Básica*) that has the purpose of providing expansion of coverage to serve populations in vulnerable situations, among others. In Brazil, the 2011 and 2017 PHC policies [20, 21] stimulated the implementation of Family Health Teams for the assistance of the riverside population of the Legal Amazon and Pantanal through two distinct types, called ‘Riverside Family Health Teams’ and ‘Fluvial Family Health Teams’. The latter work in fluvial mobile units (FMU) whose construction was also funded by the PNAB. The performance of these teams and the FMU are the subject of interest discussed in this study. The recognition of this type of assistance by the federal government is of paramount importance as it implies the transfer of financial resources to the municipalities that implement FMU, contributing to the maintenance of an activity that had previously existed in several localities before the regulation, but they were fully funded by the municipal health systems, which generated considerable financial burdens for the municipalities.

It is noteworthy that most of the municipalities where there is a demand for care in a fluvial unit have low HDI, fragile economy and, consequently, low tax collection and limited health resources [2]. Under these circumstances, the possibility of increasing health investments through federal government transfers was a key facilitator of access to health services. In addition, the locomotion of FMU implies considerable expenses with logistics and fuel, which does not occur in urban service contexts. Thus, the official acknowledgement of this alternative for delivering primary care has broadened and enhanced its relevance as a preferential way for service organization in difficult access areas, thus, analyzing the potentialities and limitations of this type of service organization is necessary [22].

However, the itinerant characteristic of these units and the difficult access to specialized health services in the cities require further analyses that show whether this model of service organization is capable of positively intervening in the health status and effectively reducing access barriers to health care and whether these healthcare practices are adequate to meet the needs of these populations.

The objective of the study was to characterize the use of health services by the Amazon rural riverside populations with the purpose of contributing to reduce access barriers to health services and ensure the right to health care.

## Methods

### Study design

The study analyzes data from the qualitative component of a broader quali-quantitative study that has been conducted in the study population. The qualitative component was developed through an ethnographic approach based on the triangulation of data sources [23]. Overall, the study should be considered a case study [23], focused on the understanding of the system of relationships established around a social practice – primary care delivered by a fluvial health unity – with the potential to infer general characteristics of the *modus operandi* of health policy designed for rural populations through FMU.

The information was obtained from observing health care routines on the FMU, whose activities are the formal dimension of care delivery, and in-depth interviews to evaluate the point of view of the professionals working on the FMU. The users were interviewed and the delivery of care was observed, focusing on pregnant women seeking antenatal care. Antenatal care was understood as a tracking condition, that is, capable of generating easily understandable information about the characteristics and results of the services provided, allowing the understanding of the interaction between users and service providers, besides enabling the development of proposals for the improvement of actions and services delivered [24].

### Participants and setting

**Fig. 1:**
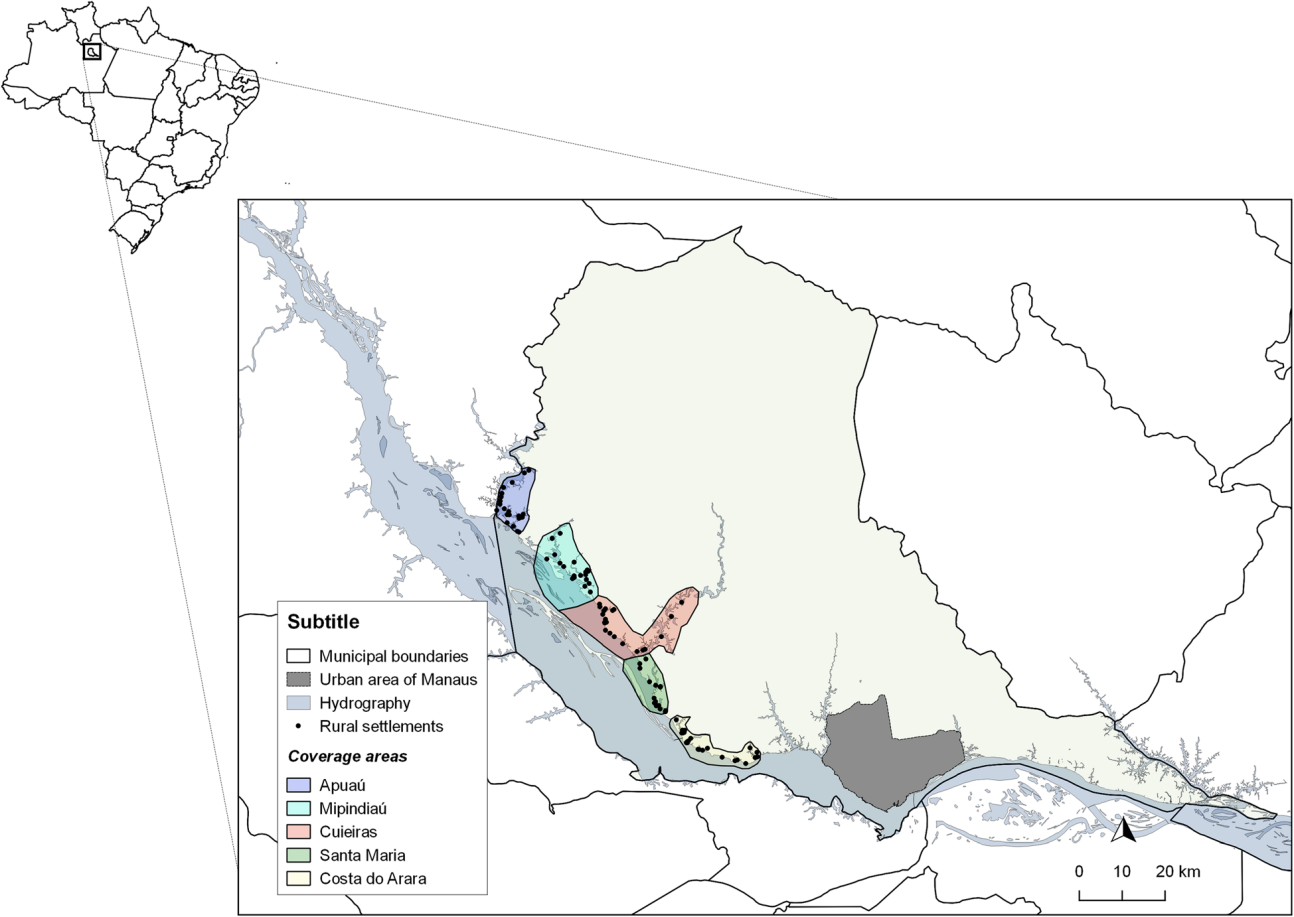
Rural settlements located on the left bank of the Negro river covered by the study

The studied population involved all professionals who had been working on the FMU for at least 1 year and the service users who live along the banks of the Negro river, in the stretch located between the municipalities of Manaus and Novo Airão in the state of Amazonas, Brazil. Thus, the study collected information from 38 rural riverside settlements, representing a total of 765 households and 2342 dwellers (Fig. 1). There were no health professionals based in the territory, except for community health workers (CHW) who are part of the Fluvial Family Health Team that intermittently serves the population on the FMU. Access to all evaluated settlements was exclusively reached by river transportation, taking up to 12 h to reach the most distant localities from the urban area of Manaus. The riverside population assessed is a rural population whose pace and lifestyle are closely related to social and environmental characteristics that impact not only the living conditions but also access to goods and services, including health services.

### Data collection

Data were collected between July 2017 and July 2019 by all the authors, using multiple strategies to gather material for qualitative analysis [25]. The collection was carried out by the simultaneous use of four current techniques in qualitative approaches: 1) the search for documents, specifically secondary official data sources, based on a profile of actions planned in the technical norms that guide the organization of PHC in Brazil; 2) systematic observation focused on service routines and daily interaction of the multi-professional team responsible for the care provided in the territory on the FMU; 3) collection of in-depth interviews carried out with health professionals with the purpose of grasping the ideas and values that surround and attribute meanings to health care and the convergence and divergence of standardized work and the work effectively carried out in the research area; 4) interviews with users of the services of FMU, residents in the territory studied, focused on understanding their perception about the care offered, and the conditions of access and use of the service. In a specific community, data collection prioritized the experiences of pregnant women concerning antenatal care received [23, 26].

Participant observation carried out on the FMU concentrated on understanding the characteristics of provision and performance of primary care on an itinerant regime. Observation of the work carried out on the FMU as a means for understanding the activities and interrelationships between health team members, users and territory, covered the various aspects of care provided during the monthly trips. Throughout the study, the researchers observed the care provided during the FMU’s trips throughout the territory, performing systematic observation in all sectors in which care was provided to users, such as in the waiting room, vaccination room, pharmacy, laboratory, dental and nursing offices, medical consultation office, social work, health education and along situational diagnosis in the territory. The activities preceding and following the FMU trip and attendance were also investigated, such as work meetings, registration of activities and procedures, scheduling and follow-up of users’ exams during the travel intervals. Due to the limitation of physical space on the mobile unit and as determined by the Municipal Health Department, the presence of one researcher per trip was allowed. With each trip lasting 10 days, there were 50 days of observation. The observation was guided by the itinerary taken by the selected patients, accompanying them and observing the care received. The selection of patients to be observed followed two procedures: 1) random selection of one out of five patients who received care on each day of the team’s work; 2) convenience sample from patients who visited sectors that had not been covered for observation through the initial strategy – this procedure aimed to ensure that all service sectors were covered by the observation.

In-depth interviews were also carried out with all 16 professionals from the primary care team who had been working on the FMU for over 1 year who provide care in the territory studied. Nurses, doctors, dentists, pharmacists, social workers and nursing technicians were interviewed by a single interviewer (MLRP), using a semi-structured questionnaire. Only one professional who had been working on the FMU for less than a year was excluded. There were no refusals. The interviews were carried out in two moments: during the FMU trip at non-service hours; and in Manaus, out of the time and place of work, aiming to eliminate any information biases introduced in the data collection performed at the workplace.

Data related to users were obtained from qualitative information obtained from dwellers by the authors when accompanying a home-based health survey, carried out during the main broader project, involving 287 households and 572 individuals. The present manuscript prioritized the information that dealt with the organization of care at the FMU and the performance of the CHW. CHW were considered as important links between the service and the community since they are the only professionals established in the territory. The users’ point of view was also evaluated in a specific community study component, mainly focusing on access to antenatal care. Community selection for the study on antenatal care was intentional, as the criterion for choosing it was the largest number of pregnant women during the fieldwork (*n* = 6), and one of the authors (PCC) spent 30 days living in the community to perform this study component. A previous observation script was not adopted, opting for the systematic noting of all events that occurred in the observed care itinerary of patients.

In all research contexts, according to the ethnographic method, field diaries were kept to systematize the experiences of the research team. The in-depth interviews were audio recorded and transcribed for further analysis of the information collected and situations observed. The decision to finalize qualitative data collection at each phase of the fieldwork was determined by the data saturation method [27].

### Data analysis

The information obtained was submitted to thematic discourse analysis [28]. The transcribed empirical material was first organized, and the central ideas and themes related to the study objectives were comprehended after thorough assessment and confrontation of the data. The main analytical categories that emerged from the field data were care model and work organization in primary care; interaction characteristics and ways of acting of the multi-professional team in an itinerant context; access and use of PHC services in rural contexts; operationalization of actions and specificities of care on the FMU.

## Results and discussion

The primary care FMU is a ship adapted for PHC, coordinated by the Rural Health District, which is one of the five health districts of the Municipal Health Department responsible for managing the network of PHC. In addition to the multi-professional teams delivering health care, the health district also has a management group responsible for the general administration of activities, workforce management, planning, organizing and assessing actions, as well as providing supplies and other logistic demands to ensure health care in rural areas where the service is performed.

The presentation and discussion of the findings followed the proposal of Cecílio for the study of evaluative processes of health care management. The author conceptualizes the management of care as the “*provision or availability of health technologies, according to the particular needs of each person, at different periods of their lives, aiming to provide well-being, safety and autonomy...*” [29]. In the proposed evaluation model, the author recognizes five dimensions of care management: individual, family, professional, organizational and systemic societal dimensions. The professional and organizational dimensions on the FMU are of special interest in this manuscript. The professional dimension is characterized as a meeting space between professionals and users and where the micropolitics of labor relations is more clearly expressed. The organizational dimension of care is forged by the technical and social division of work, representing the *locus* for teamwork, coordination of activities, interprofessional communication and care management produced in the health service. The systemic dimension – understood as the formal, institutionalized and regulated connections between health services, configuring networks or lines of care – was also addressed indirectly, as the research data identified health needs that cannot be met within the scope of PHC or within the scope of actions and services offered by the FMU.

### Care regimen, infrastructure of fluvial mobile unit and composition of the riverine primary care team

The work in the rural areas exclusively accessible by water requires the monthly locomotion of the team during the fluvial journey from Manaus to the boundary of the municipality of Novo Airão. The travel time lasts approximately 12 h to reach the first community to be served, the furthest from the departure place, as work begins at the most distant locations and ends at those closest to the urban center of Manaus. Throughout the study, the total time spent in the field was ten days per monthly trip, totaling 12 annual trips. The CHW do not travel on the FMU, as they permanently reside in the territory and are distributed among the riverside localities.

Every month, the FMU serves about 20 of the 38 localities in the coverage area on each trip, with a stay of about 4 h at each destination, which preferably occurs in densely populated settlements. As the number of existing locations is greater than those that mobile unit can visit each month, the annual schedule switches the FMU destinations to maximize healthcare opportunities for all the localities. Residents in settlements not covered by the FMU stop must travel by their own means to the docking sites of the mobile unit. Despite this important limitation, the coverage achieved by the FMU meet the official guidelines for each community to be visited “at least every sixty days” [30].

Regarding the physical structure, the FMU has a nursing office, attendance room, dental office, vaccination room, clinical analysis laboratory, pharmacy, waiting room, storeroom, sterilization room and storage warehouse for medical supplies. Support areas include health team and crew rest compartment, kitchen and dining facilities, toilets, decks, engine and electric generator rooms, fuel storage depots, cleaning supplies, and other necessary items for staff maintenance during the trip. The physical structure fully meets (and exceeds) the requirements established by the PHC policy for the FMUs [20, 21, 30, 31].

At the time of data collection two doctors, two nurses, four nurse technicians, one social worker, two dentists and two dental hygienists, one pharmacist, one biochemist, one clinical analysis laboratory assistant, one administrative professional and 18 CHW worked on the FMU, representing two riverine family health teams working simultaneously in the territory. The crew had five members.

### Care and organization characteristics of the fluvial mobile unit

The actions carried out on the FMU included medical, dental, pharmaceutical, laboratory and nursing care. Vaccination, communicable disease control with emphasis on diagnosis, treatment and control of malaria and other vector diseases, antenatal care, and other women’s health actions, such as family planning and cervical cancer screening, are offered. Actions focusing on child health, treatment and control of non-communicable chronic diseases, home visits, health education, minor surgeries and laboratory clinical analyses are also offered, along with other activities, such as anti-rabies vaccination for dogs.

CHW and community agents of endemic diseases develop tasks that are independent of the presence of the FMU such as home visits, surveillance of suspected vector diseases such as malaria, dengue and Zika, health monitoring in the territory, measurement of weight, blood pressure and glucose, guidance on health care and schedule medical and nursing appointments to be held on the mobile unit.

On the days preceding the presence of the FMU at the localities, the CHW triages people in demand for medical and dental care. On the day of attendance, the selected ones receive small pieces of paper, the so-called “tokens”, which ensure access to the doctor or dentist appointment. The bottleneck for users seeking to obtain medical care from the family health team is a well-known limitation in primary care practiced in the Amazon, and it was reported as recurrent in a previous study according to 25% of rural users who sought care in urban areas and 11% of rural users who sought care at their own home territories [3]. This finding attests to the existence of limitations in the provision of health care, either by organizational inadequacies of service or insufficient availability of professionals and consultations necessary to meet the needs of the population.

Dental appointments are scheduled by the CHW as well as previously scheduled with the dentists to follow-up on dental treatments that had been initiated during previous consultations. These appointments are considered scheduled demands. Dentistry is an area that has achieved minimum balance on the FMU between responding to spontaneous demand – associated with acute conditions and high chance of tooth extraction – and planned demand for preventive actions and restoration of decayed teeth to reduce the frequency of mutilating procedures.

Once the triage was complete, the population was invited to participate in health education activities. During the study period, it was found that the themes were in accordance with the annual calendar established by the Ministry of Health that had been passed on to the FMU team by the management professionals. Lectures on selected themes, focusing on prevention and control of cervical and prostate cancer, vaccination, suicide prevention and control of frequent diseases, were delivered during educational activities, but no intersectoral approaches were observed to improve living conditions, stimulate healthcare users to choose the themes, nor the adoption of active education methodologies to improve the quality of life and develop individual and collective autonomy of healthcare users, as recommended by the PNAB [21].

In summary, the observed health educational actions followed a pre-established script by extraterritorial official powers that stipulate, through a top-down decision-making process, the educational priorities developed on the FMU, which leaves healthcare users without any choice to explore topics of interest and interact in a horizontal relation with the team. After the lecture, users were invited by the team members to follow the pre-established order of the “tokens”, thus starting the scheduled attendance of the day shift.

Medical work was mainly based on complaint-based protocols adopted for the users seeking care, reproducing, not only on the FMU, but in the PHC network as a whole, a criticized work profile guided by the biomedical curative model that prioritizes controlling individual risks and diseases to the detriment of collective problems [32], most of them unknown by the health professionals, given the limited use of epidemiological indicators by the team. The physician’s performance acknowledges the limitation of available diagnostic resources and the short period of stay in each place requires an immediate conduct, as follow-up will only occur after a long time. Triage conducted by the CHW results in a high demand for medical consultations on each day shift, reducing the time that doctors have to focus on a more accurate assessment of health needs, evaluate determinants and risk groups in population and make an appropriate appraisal of vulnerabilities and health needs in the territory [20].

The work regime adopted on the FMU allows a large flow of users through spontaneous demand. On the other hand, the predominance of spontaneous demands limits preventive actions and home visits – advocated as one of the health care priorities in the PNAB – which is now limited to bedridden patients and emergency care due to the short time spent in each location. The imbalance in favor of spontaneous demand hinders the shared organization of the agenda, regular inclusion of home visits, provision of organized comprehensive care and monitoring of potentially severe conditions or life-threatening endemic/epidemic events in the covered territory.

“It's a little bit busy, right? As we only have one period in the community, we have to perform service without leaving anyone unattended. Thus, at the end, we do routine visits. Sometimes we even have to postpone the boat departure to another community if there are any urgent visits. In these cases, when we know about them, we always try to see them first” (Physician, Interviewee 4).

The professional’s statement indicates that the care of symptomatic patients who spontaneously seek the FMU assumes a preponderant role in daily work, making it difficult to deal with health problems that require longitudinal and/or intersectoral actions that the population does not recognize or chooses not to give them priority, given the very limited time the FMU spends in each community. It is not a matter of disregarding the users’ priorities, but rather highlighting the need to take into account the diversity of health situations in the territory and deal with problems that are not evident to the population, either due to unspecific clinical expression or the long latency time, as it is the case of non-communicable chronic diseases. This does not mean that the healthcare user should be blamed for spontaneously seeking the mobile unit, but rather understanding that there are flaws in the organization of the service, once the recognition of problems in the territory is insufficient to improve the schedule of activities and deal with issues that could have been solved before becoming a reason for spontaneous demand. In addition, the service may be able to reorganize routines in order to increase the allocation of professionals’ time to include prevention, or even train and qualify the CHWs so they could play a broader role in the communities.

Among the consequences of the imbalance between spontaneous demand and planned demand is the scant attention paid, for example, to eating and nutritional disorders. Although the literature has pointed to a significant reduction in undernutrition in Brazilian childhood, the prevalence of child undernutrition in the Amazon remains a concern as 3.3% of non-indigenous children under 5 years in the North have weight deficit, as opposed to 1.9% of non-indigenous low-weight Brazilian children for the equivalent age [33–35]. Undernutrition is more prevalent among indigenous children living on indigenous (rural) lands in the northern region and 11.4% of them present a weight deficit. The short stature for age is equally high among indigenous children (25% on indigenous lands as a whole) and 40.8% among children under five residing in indigenous lands in the North. In contrast, for non-indigenous children in Brazil, the prevalence of short stature for age is 7.0% [36–38]. The indicators found in the child population coexist with overweight and obesity among adults, reaffirming the relevance of nutritional disorders as a public health problem [33].

Although the interviewees claim to consider the nutritional problem as relevant, recognizing the problem seems to have little effect on the daily work of the team, as there were no activities on the agenda for this purpose during the data collection period, nor the offer of planned and regular monitoring actions on the growth and development of children or prevention and monitoring of overweight and obesity among adults. In a healthcare model guided by spontaneous demand, health issues such as discrete (e.g. chronic undernutrition) or late clinical outcomes (e.g. overweight and obesity) only becomes priority when they move from subclinical to evident or uncomfortable conditions that cause enough discomfort to encourage users to seek medical care.

Nursing care is more flexible in managing spontaneous demand and solving health problems at their level of professional competence. However, the nursing consultation also works as an access to medical care if the nurse identifies an immediate medical need. The range of nursing responsibilities is wide, and nurses are responsible for both clinical and administrative tasks that include systematization of service health records, report writing, notification of diseases and control of supplies. They also help prevent and control diseases of epidemiological relevance such as hypertension, diabetes, cervical and breast cancer, tuberculosis and other communicable diseases, as well as being responsible for activities aimed at specific population groups, such as women and children, who participate in antenatal, contraception and vaccination actions.

The breadth of the nurses’ tasks provides them a comprehensive view of the health inequalities prevailing in the territory and the need to organize the team’s activities to face these problems. This characteristic of the nursing practice might also be influenced by the nurses’ turnover rate, which is lower than that of doctors.

“We know all the patients, we know everybody: who is well and who is not […] first because they do not have access to all government services and because their purchasing power is [low], as they do not have access to health care. They are special people because they are deprived of health, education and security… they are vulnerable. So, we should consider them from a different point of view because their perception of the world and life is different. We have conducted studies in some communities and found a high rate of diarrhea due to the lack of treated water [...] On the other hand, there is no government restructuring actions to help people organize, for example, a home garden, so they have the means to develop their own talents, improve their income and quality of life. So, all this has a cascade effect that reflects on health. We only take care of health, but we have to pay special attention to other things because we deal with socially excluded people” (Nurse, Interviewee 5).

In addition to the great geographical distances, scarcity of employment and other sources of work in the study territory limit the opportunities for income improvement, making the possible commercialization of rural production difficult, for example, since access to the nearest cities requires days of travel and there are no means of transportation that ensure production flow [39]. Overall, the scenario of significant social vulnerability cannot be solved or mitigated through curative care that is usually offered by the PHC network in Brazil.

“There are things we do that go far beyond technical care. It is common patients only has enough fuel to arrive [at the unit], but no means to return home. Then we find a way to help them get back… they never leave without a solution to their problem, be it a simple or a more complex problem” (Nurse, Interviewee 5).

“Given the ties we have with communities, our activities extend over the course of a month and years. We don’t only get involved in the [healthcare] organization in the rural health district, at meetings, or on the day of the trip. I work with antenatal and preventive [screening to detect cervical cancer]. I must check, in Manaus, if the results of the women's exams are available. I end up getting involved; I contact the laboratory to see if the exams were really treated with due importance; if the analysis was done and sent back because then we can deliver a satisfactory result to the women” (Nurse, Interviewee 5).

In the absence of guidance or specific regulations from the Department of Health to guide the work on the FMU, professionals have been adapting routines, usually designed for urban areas, to the singularities of the rural space where they work. One of them is related to the understanding of the living conditions of the population, which is an even more urgent need considering that the professionals who work on the mobile unit came from large cities, since part of them have migrated from other regions of Brazil to the Amazon and have limited knowledge of the way of life of the rural populations under their responsibility.

In view of this difficulty, the team developed and began to apply an information gathering instrument with the purpose of understanding the way of life, housing patterns, demographic and socioeconomic profile of families living in their area, improving the definition of the territory [20]. Data collection, which is not recognized by all team members, is seen as an efficient strategy to strengthen the bond with the population and acknowledge the health needs that are not addressed during the routine tasks of the team. It also provides tools for intersectional approaches that favor overcoming the persistent biomedical model, as exemplified by the following statement:

“At the end of the research survey, we will provide direct care to the most precarious situations, right? They used to be afraid to report family income, but nowadays they us tell everything. So [the information collected] goes from vaccinations to who lives in the house; if there are any unsolved problems that have not been dealt with, and so on. Because [during the interview] there is a lot of information that we can't verify during attendance, for example, cases of violence against women, children or sexual abuse. It's a common problem in communities and it's a new focus that the team has been paying attention to” (Physician, Interviewee 4).

In addition, it creates new possibilities for the interaction between team members that go beyond the usual health professional hierarchies.

“Socioeconomic research and planning have been very interesting. Everyone participates; everyone speaks up and has the same power of opinion. In research nobody has unique decision-making power and that is a pretty cool thing. Management professionals don’t participate, but they should participate and accept the research. But this is our own planning, just the team's; management is separate… they make decisions in isolation and want to shove them down our throats without our participation. It is a pity, because we really know all about the community. Those who are there [in the community] really know what is going on; we know the reality, the real facts and not just facts on paper [from reports]” (Nurse, Interviewee 1).

### Interaction and modus operandi of the multidisciplinary team

The interaction between team members is also one of the specificities of FMU’s performance, with significant intervention in the work organization that provides care in the health unit. Authors such as Peduzzi [40] distinguish health teams that work with what is called a “team group”, whose main characteristic is the juxtaposition of actions developed by different professionals. The second type mentioned by the same author is the so-called “integration team” whose work alignment favors the interprofessional articulation of activities, which is the model recommended for PHC. The interviewed professional’s testimony evidences the coexistence of the two types of interaction: the horizontality in the relations between the team members in the territory, as opposed to the verticality of the relations established by the managers of the Rural Health District.

“We have a meeting on every trip before the boat leaves, but we can't speak up as much as when we are working as a team during the trip. When we stop at a community, we investigate what the community's [lifestyle] is. We get to know the age of the population; what are the weaknesses of families, for example. But before that, the team meets to set up goals, seek and solve pending healthcare issues. The nurses usually lead the meetings, but the whole team participates, no matter if you are the expert in dermatology, a technical nurse or any other professional. During the meeting everyone can speak up. What I think is really cool is that everyone respects each one’s field. If you work in the pharmacy, you have to lead the discussion on that subject […] We know it's not easy to travel for so many days with different personalities, but it's all well-organized. When I arrived, everything was already established, but the team says it was not easy to move from healthcare task force to a family health strategy. I think everyone is to be congratulated … we realize that it was the intention of the team: they, all the professionals who have been here, started organizing service flows. So, for me, the organization workflow is complete” (Physician, Interviewee 4).

The report points to a specific dimension of professional interactions at FMU where embarked work establishes a coexistence regimen that surpasses the professional plan and assumes a personal character resulting from the intimate and prolonged coexistence of people who work, eat and sleep in the same environment, share labor difficulties and risks of hostile nature over 2 weeks per month for years.

The role of the CHW is marked by the fact that these represent the only continuous source of care in the travel intervals. As a result, most of their activities are carried out autonomously and are largely unknown to the team. Team members see this feature positively:

“Community health workers are our connection to community. So, in our absence, the health workers collect information and transmit it to us when we arrive. That's why I say there is better interaction [with the community] because they already know what we will ask them” (Nurse, Interviewee 1).

“We give community health workers a lot of freedom because they work on a daily basis with the community and they know the reality. So, whenever someone needs early attendance [a consultation], we don't restrict the number of patients … we see whomever needs attendance. We have a limit of vacancies [for daily service] that is always extrapolated. In addition [at the end of the day shift] we see pregnant women and urgencies. But if there are a lot of teenage pregnancies, we always ask them [community workers] to try to call them. Try to bring call the girls who are of childbearing age to give them guidance. But this is well organized, and we do not need to interfere too much, and we soon see them going to the consultation” (Physician, Interviewee 4).

Although CHW know the families of primary care users personally, their decision-making on who should receive priority care is only based on common sense, as they have no training or professional experience that enable them to recognize and classify situations of risk and vulnerability. Thus, their intervention for increasing medical appointments, including additional new patients in addition to those who are already scheduled, tends to direct the time the team remains in the community to carry out consultations resulting from spontaneous demand instead of being able to focus on health promotion and preventive activities.

Nunes et al. [41] emphasize the different types of activities carried out by the community workers, that are linked to the demands and tasks established by the health system. On the other hand, the CHW have to respond to the demands arising from the power relations waged at the community level, which are barely recognized by other health team members. The scheduling of appointments for the FMU derived from the triage conducted by the CHW may express not only the need for medical care, but also responses to political and kinship commitments with the families receiving care, ignoring other problems whose severity the community worker cannot perceive or patients who do not perceive themselves as priorities in the network of influential relationships in the community.

The romanticized view that interviewees have of this professional’s work contrasts with observation data and the viewpoint of healthcare users. Users recurrently criticize the power enjoyed by the CHW who decide who will be granted access to medical appointments. According to the service users, they tend to favor family members or political partners when distributing tokens that ensure access to medical appointments during the stay of the FMU instead of those whose disease symptoms require care. The observation also found that the greater or lesser proximity between the main housing clusters, the residence of the community worker and the home of user seeking care also have positive or negative influences on the access to health care on the FMU.

The definition of the tasks of the CHW also lacks clarity, particularly during FMU travel intervals. No therapeutic projects shared between them and the rest of the health team, whose continuity would be ensured in the absence of other professionals, were observed. Rural health district management, on the other hand, has adopted a management arrangement that makes it difficult to integrate community workers with other FMU team members. The rural health district directly and separately from the rest of the team organizes the work of the CHW. This option may be administratively rational, but in terms of the organization of care in the territory, it makes the exchange of experiences and the collective development of action plans for addressing problem situations in the territory unfeasible as well as hindering the continuity of monitoring actions that could be carried out by community workers during the FMU travel intervals.

### Access and use of primary health care services in the rural context

Considering the geographical distances and the long travel time to travel to and from the places of residence of FMU-assisted families to health services based only in the nearest cities, the service plays a crucial role in ensuring access to PHC, either by effectively accommodating spontaneous demand by directly offering primary care actions, or by enabling the scheduling of specialized services, which, in the Brazilian health system, depends on a regulatory scheduling of examinations and consultations upon medical request.

Among the actions that enhance access to health care, we selected the analysis of two of them. The first one is the pharmaceutical and laboratory assistance offered on the FMU. In Brazil, primary care units usually do not have comprehensive pharmaceutical assistance in their facilities. The studied FMU has a pharmacy service and a wider range of medication than the one usually available in the PHC network. In addition, it has been able to regularly meet the needs of healthcare users covered by the unit and provide medication that would only be available at specialized units, such as psychotropics.

The mobile unit also has a clinical analysis laboratory that performs the demanded analysis and provides the results on the same day shift the material was collected. Such uniqueness of the FMU confirms the need to increase the effectiveness of the service, ensuring access to diagnostic resources during the presence of the team in the territory, facilitating the immediate adoption of protocols required to solve problems and reducing patient referrals to the city, where the difficulties of access to consultations and exams for users coming from rural areas significantly increase. The implementation of the clinical analysis laboratory also reflects the broadening of the range of actions of the FMU compared to terrestrial primary care units, since most of them do not have laboratory facilities, requiring longer waiting times for exam results.

The assessment of the effectiveness of installed capacity of the FMU is ongoing and the data collected have not yet enabled an overall assessment of the activities on the mobile unit. Partial results, such as the assessment of antenatal care, indicate good performance of this type of action. The technical standards regulating antenatal care in Brazil recommend that it should begin preferentially in the first trimester of pregnancy, up to the 20th gestational week. A total of at least seven consultations, including the postpartum consultation up to 7 days after delivery, is also recommended. Pregnant women should also receive dental follow-up, laboratorial, food and nutritional guidance, iron supplementation and vaccination when necessary. Laboratory exams are recommended preferably during the first trimester of pregnancy and repeat laboratory testing should be requested according to needs.

The observation of antenatal care offered by the FMU has highlighted a number of adjustments the team has made to these routines to speed up the consultation of pregnant women on the mobile unit and optimize their time spent on it, in addition to meeting the specificities of the rural context. The analysis of the adequacy of the physical structure showed that there are 94.6% of the items required for antenatal care. This percentage is much higher than that found in the PHC network in Brazil (26%) and for the northern region, whose infrastructure is only 18% adequate for antenatal care [12].

All required medications and antenatal laboratory exams were available on the FMU and 94.6% of pregnant women eligible for vaccination doses were covered. Of the registered pregnant women, 98.9% of them performed the recommended laboratory exams; it is noteworthy that the VDRL test was performed on 100% of pregnant women registered in the mobile unit, in contrast to 69.5% in the PHC network of the Brazilian Amazon [11]. A total of 92.5% of pregnant women that receive care on the FMU started antenatal care up to the 20th week of pregnancy (Kessner index). However, when a most demanding indicator (Kotelchuck index) is used, which advocates admission up to the 12th week, this percentage drops to 63.4%. This percentage is lower than that found for pregnant women from the Brazilian Amazon (rural and urban) who 74.4% started antenatal care during the first trimester of pregnancy.

Compliance with the seven consultations recommended for pregnancy was only observed for 62.4% of pregnant women, which is far from the 73% found for Brazil [41]. The commitment to this goal on the FMU is associated with the difficulty of offering postpartum consultations during first week after delivery, a condition attended only by 46.7% of pregnant women registered at the unit. The achievement of the goal is compromised by the great distance between the pregnant woman’s home and the place of delivery in hospitals located in urban areas. By the time the postpartum woman returns to her home and can be reevaluated by the itinerant team, the time interval has already exceeded the recommended one.

Clinical care was offered to 100% of pregnant women; however, the set of recommended actions for this assessment item was low. The highest percentage observed was the record of gestational age (86%), weight and height of the pregnant woman (55.9%). The lowest percentages recorded were auscultation and evaluation of fetal movement (37.6%) and pregnancy risk rating (4.3%). These are undesirable limitations, but the results achieved on the FMU are equivalent to, or even surpass in some respects, those measured for the antenatal care in the PHC network for the entire Brazilian territory [42]. In the study period, no cases of neonatal syphilis were recorded, and no vertical transmission of HIV was observed in pregnant women registered at the service.

The observation evidenced problems in the management of information related to the procedures performed: the FMU team did not keep a copy of the pregnancy record book (“mirror card”) nor had any other means of recording antenatal care, except for medical records. The procedures for several consultations were not recorded because the medical records moved among different sectors on the same day shift and were not always available for the professional to write down the information of the consultation. In addition, the record is written on various sheets of the medical record, making it difficult to globally assess antenatal care. Overall, in spite of the difficulties pointed out – management being the one with the highest incidence of problems –antenatal care offered by the FMU is comparable in terms of quality and effectiveness with the results observed in urban primary care units, which is suitable for normal pregnancies without clinical complications despite the itinerant characteristic of the mobile unit. However, strategies are needed to increase the registration of pregnant women at the beginning of pregnancy and provide means to ensure the management of obstetric complications that may occur in the territory during the absence of the FMU team [9].

### Focus on the healthcare user: an advance in access to primary health care, but logic replicates the urban context

Before the implementation of the fluvial PHC strategy in the territory, healthcare users mentioned difficulties accessing health services, which were only recalled regarding actions against malaria in the region. Given the impossibility of performing a diagnostic test to confirm the disease, medications were distributed to the population, which were to be used if any malaria symptoms were noticed. At that time there was no regular transportation and canoes were the main means of transport to the nearest urban areas. This implies that some diseases were only treated with herbal medicines, or even through self-medication using pills sold by river traders. If lucky, a philanthropic boat could offer minimal medical and dental care, otherwise “*if the disease were a killer, I would have died*” (Service user). An additional file shows the transcript of all the statements presented in the article in the original language [see Additional file 1].

Thus, the regular availability of itinerant services, such as the FMU, changes the users’ relationship with the health system. From “circumstantial aid” to a monthly event in the community routine, health actions are becoming increasingly tangible for users. The community appropriates the change in the logic of care delivery, fighting for it and allowing the health service to be integrated to the territory. On the day of monthly service of the FMU, the community gains new perspectives. Intra-community reciprocal relations are strengthened by the solidarity of local residents with relatives and neighbors who live in nearby localities (by offering them a ride on larger boats, gas sharing and hosting people at their homes) and trade relations are also established (selling food and drinks). People yearn for this day, participate of the ‘screening ritual’ a few days earlier and dress up for a special meeting – with ‘relatives’ and the ‘urban other’.

This ‘urban other’, although foreign to the rural world and bursting with biomedical prescriptions, also gains new perspectives with the continuity of the service offered. Health professionals who allow themselves to establish care ties with users, not treating them as mere clinical cases, but as equal human beings, respecting subjectivities and peculiarities, gain respect and acknowledgement. The population rewards them by consolidating relationships or even by occasionally offering of fruit, eggs or vegetables. The ties created and fostered with each new contact enable the combination of technical-scientific knowledge with the established traditional conceptions about the health-disease process, favoring new arrangements from this understanding [22]. But this encounter can also reject health professionals who are unwilling to transpose the urban logic of health care or even understand the subjectivity of individuals who seek care. In some cases, community members only seek the care of those with whom they feel affection and know that they will be listened to.

Overall, the establishment of continuous service offered to these populations has considerably increased access to PHC. The presence of primary care in the daily life of the population has provided preventive and biomedical curative actions, without suppressing traditional practices and the use of herbal medicines that are part of the local culture. However, there are still barriers to access continued care, planned according to local specificities, as well as difficulties to access specialized services.

## Conclusions

The implementation of the FMU represents an advance in terms of accessibility to PHC, with the potential to focus work on the territory to change the healthcare model, which is less biomedical and more interactive. However, the short length of stay in each community, limited involvement of the CHW and a healthcare model focused on spontaneous demand do not improve access to reach those who live in restricted geographic areas and the most vulnerable and in need of care. This ‘mismatch between worlds’ in the healthcare organization uncritically replicates the routines adopted in the daily healthcare of services located in urban spaces, proving itself inadequate in the provision of assistance strategies capable of mitigating social and health inequalities faced by the users. Thus, inconsistencies and inadequacies persist in the ways of recognizing the particular social scenarios in rural Amazonia, not contributing to overcoming their socio-political and economic invisibility and social exclusion from the national priorities.

## Supplementary information

**Additional file 1.**

## Data Availability

The data that support the findings of this study are available from the corresponding author upon reasonable request. The data are not publicly available due containing information that could compromise research participant privacy/consent.

## Abbreviations

CHW: Community Health Workers
FMU: Fluvial Mobile Unit
HDI: Human Development Index
PHC: Primary Health Care
PNAB: National Policy for Primary Health Care
