# Supplementary material for: Barriers to access and organization of primary health care services for rural riverside populations in the Amazon
Source: Int J Equity Health. 2020 Jul 31;19:54. doi: 10.1186/s12939-020-01171-x (PMC7394681; doi:10.1186/s12939-020-01171-x)
Supplement: Supplementary file 1 — Additional file 1. [file 12939_2020_1171_MOESM1_ESM.docx]

**Transcript of the statements presented in the article in the original language (Portuguese)**

“It's a little bit busy, right? As we only have one period in the community, we have to perform service without leaving anyone unattended. Thus, at the end, we do routine visits. Sometimes we even have to postpone the boat departure to another community if there are any urgent visits. In these cases, when we know about them, we always try to see them first” (Physician, Interviewee 4).

“We know all the patients, we know everybody: who is well and who is not [...] first because they do not have access to all government services and because their purchasing power is [low], as they do not have access to health care. They are special people because they are deprived of health, education and security… they are vulnerable. So, should consider them from a different point of view because their perception of the world and life is different. We have conducted studies in some communities and found a high rate of diarrhea due to the lack of treated water [...] On the other hand, there is no government restructuring actions to help people organize, for example, a home garden, so they have the means to develop their own talents, improve their income and quality of life. So, all this has a cascade effect that reflects on health. We only take care of health, but we have to pay special attention to other things because we deal with socially excluded people” (Nurse, Interviewee 5).

“There are things we do that go far beyond technical care. It is common patients only has enough fuel to arrive [at the unit], but no means to return home. Then we find a way to help them get back… they never leave without a solution to their problem, be it a simple or a more complex problem” (Nurse, Interviewee 5)

*“Fica um pouquinho corrido, né? Porque como a gente só tem um período na comunidade e temos que realizar esse atendimento sem deixar ninguém descoberto. Com isso, no tempo final que sobra é que fazemos as visitas de rotina. As vezes temos até que atrasar a saída do barco para outra comunidade se tiver alguma visita de urgência. Nesses casos, quando a gente sabe sempre procura ir primeiro” (Médico, Entrevistado 4).*

*“Nós conhecemos todos os pacientes; conhecemos todo mundo: quem está bem e quem não está [....] Começa porque eles não têm acesso a todos os serviços do poder público e também pelo [baixo] poder aquisitivo, pois eles não têm como acessar os cuidados. São pessoas muito especiais, porque são carentes de saúde, de educação, de segurança; são vulneráveis. Então a gente tem que ter um olhar diferenciado porque a percepção deles de mundo e da vida aqui, é diferente. Já fizemos alguns estudos e em algumas comunidades verificamos alto índice de diarreia devido à falta de água tratada […] Por outro lado não tem ação estruturante por parte do poder público para que essa pessoa tenha, por exemplo, uma horta familiar, para que tenha meios para desenvolver os próprios talentos, para melhorar a renda e a qualidade de vida. Então, tudo isso gera um efeito dominó que vai se refletindo na saúde. Nós só cuidamos da saúde, mas temos que dar atenção especial às outras coisas porque lidamos com pessoas excluídas” (Enfermeira, Entrevistado 5).*

*“Tem coisas que fazemos que vão muito além do atendimento meramente técnico. Não é raro que um paciente só tenha conseguido combustível para chegar para o atendimento [na unidade], mas não tenha como voltar para casa. Aí a gente já dá um jeito de ajudar ele a voltar, para que ele nunca saia sem uma solução para o problema dele, seja de uma forma mais simples ou seja de uma forma mais complexa” (Enfermeira, Entrevistado 5)*

“Given the ties we have with communities, our activities extend over the course of a month and years. We don’t only get involved in the [healthcare] organization in the rural health district, at meetings, or on the day of the trip. I work with antenatal and preventive [screening to detect cervical cancer]. I must check, in Manaus, if the results of the women's exams are available. I end up getting involved; I contact the laboratory to see if the exams were really treated with due importance; if the analysis was done and sent back because then we can deliver a satisfactory result to the women” (Nurse, Interviewee 5)

“At the end of the research survey, we will provide direct care to the most precarious situations, right? They used to be afraid to report family income, but nowadays they us tell everything. So [the information collected] goes from vaccinations to who lives in the house; if there are any unsolved problems that have not been dealt with, and so on. Because [during the interview] there is a lot of information that we can't verify during attendance, for example, cases of violence against women, children or sexual abuse. It's a common problem in communities and it's a new focus that the team has been paying attention to.” (Physician, Interviewee 4).

“Socioeconomic research and planning have been very interesting. Everyone participates; everyone speaks up and has the same power of opinion. In research nobody has unique decision-making power and that is a pretty cool thing. Management professionals don’t participate, but they should participate and accept the research. But this is our own planning, just the team's; management is separate… they make decisions in isolation and want to shove them down our throats without our participation. It is a pity, because we really know all about the community. Those who are there [in the community] really know what is going on; we know the reality, the real facts and not just facts on paper [from reports]” (Nurse, Interviewee 1)

*“Pelo vínculo que nós temos com as comunidades nossas atividades se estendem ao longo do mês e dos anos. Não é só no distrito, na reunião ou no dia da viagem que a gente se envolve com a organização [do cuidado]. Eu que trabalho com o pré-natal e com o preventivo [screening para detecção de câncer de colo de útero] tenho que ver, lá em Manaus, se saiu o resultado dos exames das mulheres. Eu acabo me envolvendo, procurando contato com o próprio laboratório para ver se realmente foi dada a devida importância; se o exame foi feito; e se foi enviado de volta, né? Porque aí a gente já leva um resultado satisfatório para a mulher” (Enfermeira, Entrevistado 5)*

*“No final do todo aquele levantamento de pesquisa a gente vai direcionar o atendimento para o que está mais precário né? Antes eles ficavam com medo de informar a renda da família, mas hoje em dia eles contam tudo. Então [a informação coletada] vai desde a parte de vacinação; quem mora na casa; se tem algum problema que a unidade não vem atendendo e assim vai. Porque [na entrevista] se encontra muita coisa que no atendimento a gente não consegue verificar. É o caso de violência contra a mulher, contra as crianças e abuso sexual. É um problema com índice muito elevado nas comunidades e isso é um novo foco que a equipe está querendo” (Médico, Entrevistado 4).*

*“Tem sido muito legal essa pesquisa socioeconômica e esse planejamento. Todos participam e todos têm a sua opinião e todos tem a mesma voz; todos tem o mesmo poder de opinar. Ninguém tem aquele poder decisório único e isso foi uma coisa bem legal. Quem não participa, mas deveria participar e acatar a pesquisa é a gerência. Mas esse é um planejamento só nosso, só da equipe; a gerência fica muito à parte, toma as decisões de forma isolada e quer enfiar para gente goela abaixo, sem a nossa participação. É uma pena, porque nós temos um olhar real. O olhar real é de quem vai lá; de quem conhece; de quem tem conhecimento da realidade, do fato real e não só de papel” (Enfermeiro, Entrevistado 1)*

“We have a meeting on every trip before the boat leaves, but we can't speak up as much as when we are working as a team during the trip. When we stop at a community, we investigate what the community's [lifestyle] is. We get to know the age of the population; what are the weaknesses of families, for example. But before that, the team meets to set up goals, seek and solve pending healthcare issues. The nurses usually lead the meetings, but the whole team participates, no matter if you are the expert in dermatology, a technical nurse or any other professional. During the meeting everyone can speak up. What I think is really cool is that everyone respects each one’s field. If you work in the pharmacy, you have to lead the discussion on that subject [...] We know it's not easy to travel for so many days with different personalities, but it's all well-organized. When I arrived, everything was already established, but the team says it was not easy to move from healthcare task force to a family health strategy. I think everyone is to be congratulated… we realize that it was the intention of the team: they, all the professionals who have been here, started organizing service flows. So, for me, the organization workflow is complete” (Physician, Interviewee 4)

“Community health workers are our connection to community. So, in our absence, the health workers collect information and transmit it to us when we arrive. That's why I say there is better interaction [with the community] because they already know what we will ask them.” (Nurse, Interviewee 1)

*“Toda viagem nós temos uma reunião antes do barco sair, mas nesta a gente não consegue ter tanta voz como quando a gente está em equipe durante a viagem. Quando paramos numa comunidade a gente faz um levantamento para ver qual é o estilo [de vida] da comunidade. Saber qual a idade da população, quais as fragilidades das famílias, assim né. Mas antes disso a equipe se reúne para traçar metas, ver e resolver as pendências do atendimento. Geralmente quem lidera a reunião são as enfermeiras, mas toda equipe participa, não importa se você é o técnico em dermatologia, se você é o técnico em enfermagem ou qualquer outro profissional. Durante a reunião todo mundo tem a mesma voz. O que eu acho muito legal é que cada um respeita sua área, se você é da farmácia, você é que tem que conduzir a discussão daquele assunto [...] A gente sabe que não é fácil viajar tantos dias com personalidades diferentes, mas é tudo muito organizado. Quando eu cheguei já encontrei tudo estabelecido, mas a equipe conta que não foi fácil passar de um atendimento que funcionava como um mutirão para uma estratégia de saúde da família. Eu acho que assim todos estão de parabéns que a gente percebe que foi o intuito da equipe: eles, todos os profissionais que já passaram por aqui, foram organizando os fluxos de atendimento. Então para mim o fluxo de organização do trabalho é muito completo” (Médico, Entrevistado 4)*

*“O ACS [agente comunitário de saúde] é o elo nosso com a comunidade. Então na nossa ausência é o ACS que tem que colher as informações para passar quando a gente chega no dia do atendimento. Por isso que eu falo que tem uma interação melhor [com a comunidade] porque o ACS já sabe tudo que a gente já vai perguntar”. (Enfermeira, Entrevistada 1)*

“We give community health workers a lot of freedom because they work on a daily basis with the community and they know the reality. So, whenever someone needs early attendance [a consultation], we don't restrict the number of patients… we see whomever needs attendance. We have a limit of vacancies [for daily service] that is always extrapolated. In addition [at the end of the day shift] we see pregnant women and urgencies. But if there are a lot of teenage pregnancies, we always ask them [community workers] to try to call them. Try to bring call the girls who are of childbearing age to give them guidance. But this is well organized, and we do not need to interfere too much, and we soon see them going to the consultation” (Physician, Interviewee 4).

“if the disease were a killer, I would have died” (Service user).

*“A gente dá muita liberdade para os ACS porque eles que estão no dia a dia da comunidade e conhecem a realidade. Então, sempre quando eles precisam encaixar alguém [na consulta] a gente não coloca limite, atende o que for necessário. Nós temos um limite de fichas [para atendimento diário] que sempre é extrapolado. Além disso [no final do turno] a gente atende as gestantes e as urgências. Mas se tá tendo muita gravidez na adolescência a gente sempre solicita para eles [os ACS]: – Olha tenta chamar mais. Tenta trazer para consultar as meninas que estão em idade fértil, para orientar. Mas isso é bem organizado e a gente não precisa interferir tanto e logo vê que realmente elas vão para o atendimento” (Médico, Entrevistado 4).*

*“se a doença fosse de morrer, morria mesmo” (Usuário).*
